# Nuclear accumulation of UBC9 contributes to SUMOylation of lamin A/C and nucleophagy in response to DNA damage

**DOI:** 10.1186/s13046-019-1048-8

**Published:** 2019-02-11

**Authors:** Yunong Li, Xiuxing Jiang, Yanhao Zhang, Ziyi Gao, Yanxia Liu, Jinjiao Hu, Xiaoye Hu, Lirong Li, Jingshan Shi, Ning Gao

**Affiliations:** 1 College of Pharmacy, Army Medical University, Chongqing, 400038 China; 2 Key Laboratory of Basic Pharmacology of Ministry of Education and Joint International Research Laboratory of Ethnomedicine of Ministry of Education, Zunyi Medical University, Zunyi, China; 3 Greater Philadelphia Pharmacy, Philadelphia, USA

**Keywords:** Nucleophagy, UBC9, SUMOylation, Lamin A/C, LC3

## Abstract

**Background:**

Macroautophagy (hereafter referred to as autophagy) is an evolutionarily conserved intracellular mechanism for lysosomal degradation of damaged cellular components. The specific degradation of nuclear components by the autophagy pathway is called nucleophagy. Most studies have focused on autophagic turnover of cytoplasmic materials, and little is known about the role of autophagy in the degradation of nuclear components.

**Methods:**

Human MDA-MB-231 and MCF-7 breast cancer cell lines were used as model systems in vitro. Induction of nucleophagy by nuclear DNA leakage was determined by western blot and immunofluorescence analyses. The interaction and colocalization of LC3 and lamin A/C was determined by immunoprecipitation and immunofluorescence. The role of the SUMO E2 ligase, UBC9, on the regulation of SUMOylation of lamin A/C and nucleophagy was determined by siRNA silencing of UBC9, and analyzed by immunoprecipitation and immunofluorescence.

**Results:**

DNA damage induced nuclear accumulation of UBC9 ligase which resulted in SUMOylation of lamin A/C and that SUMOylation of this protein was required for the interaction between the autophagy protein LC3 and lamin A/C, which was required for nucleophagy. Knockdown of UBC9 prevented SUMOylation of lamin A/C and LC3-lamin A/C interaction. This attenuated nucleophagy which degraded nuclear components lamin A/C and leaked nuclear DNA mediated by DNA damage.

**Conclusions:**

Our findings suggest that nuclear DNA leakage activates nucleophagy through UBC9-mediated SUMOylation of lamin A/C, leading to degradation of nuclear components including lamin A/C and leaked nuclear DNA.

**Electronic supplementary material:**

The online version of this article (10.1186/s13046-019-1048-8) contains supplementary material, which is available to authorized users.

## Background

Autophagy is a lysosome-dependent degradation pathway that is activated by stressful situations such as starvation, oxidative stress, and DNA damage and also has a pivotal role in the maintenance of nuclear and mitochondrial genomic integrity [1, 2]. A selective form of autophagy, known as nuclear autophagy (nucleophagy), mediates the degradation of nuclear material [3, 4]. However, the role of nucleophagy in degrading nuclear constituents is poorly understood. Several studies showed that certain nuclear components are nucleophagic targets [5–10]. The nuclear lamina is a fibrillar network located inside the nuclear envelope whose major components are the four nuclear lamin isoforms: lamins A/C, B1, B2, and their associated proteins [11]. The nuclear lamina provides the nucleus with mechanical strength and regulates higher-order chromatin organization, modulating gene expression and silencing [11–13]. Recent studies show that nucleophagy plays a critical role in degrading nuclear constituents including nuclear lamins [4, 6, 8]. Dou Z, et al. discovered a specific interaction between LC3 and lamin B1 in the nucleus in response to DNA damage, and this LC3-lamin B1 interaction mediated degradation of lamin B1 and other nuclear components, leading to nucleophagy [5, 11]. Park YE, et al. reported that mutations in the genes encoding A-type lamins (lamin A/C) and the nuclear membrane protein, emerin, cause structural deformations in the nuclear envelope resulting in the induction of nucleophagy [8].

Recent studies reveal that post-translational modification of proteins with small ubiquitin-like modifiers (SUMO), known as SUMOylation, plays an essential role in the regulation of many essential cellular activities including a variety of nuclear processes such as chromatin organization, transcription, nuclear transport, genome integrity and DNA repair [14–19]. SUMOylation of proteins can mediate novel protein-protein interactions with other proteins containing SUMO-interacting motifs (SIMs) [20]. Similar to the ubiquitination pathway, SUMOylation is catalyzed by three key enzymes: SUMO E1 (the heterodimer SAE1 and SAE2), SUMO E2-conjugating enzyme (UBE2I/UBC9), and several E3 ligases [21, 22]. Recent studies indicated that lamin A/C could be a target of SUMOylation. UBC9 conjugates SUMO1 to target proteins and interacts with lamin A/C [23–25]. However, the regulatory mechanism of UBC9-mediated SUMOylation of lamin A/C in nucleophagy remains largely unknown.

In the present study, we showed for the first time that UBC9-SUMOylated lamin A/C promotes the interaction between the autophagy protein LC3 and the nuclear lamina protein lamin A/C in response to DNA damage. Through this interaction, nucleophagy specifically mediates destruction of nuclear lamina, leading to degradation of nuclear components including lamin A/C and leaked nuclear DNA. Our findings suggest that in response to cancer-promoting stress like DNA damage, autophagy degrades nuclear material to block tumorigenesis. Our research has opened up a new direction in studying the functional role of autophagy in the nucleus and in tumor suppression.

## Materials and methods

### Cell culture

MDA-MB-231, MCF-7, A549, HepG2 and DU145 were obtained from the American Type Culture Collection. Cells were cultured in DMEM (Gibco), RPMI 1640 (Gibco) and MEM (HyClone) medium supplemented with 10% fetal bovine serum (FBS; Gibco) at 37 °C in 5% CO_2_. 293FT cells (Invitrogen, R700–07) were cultured in DMEM supplemented with 10% FBS, 0.5 mg/ml G418 (Sigma Aldrich, A1720), 4 mM L-glutamine, 0.1 mM MEM nonessential amino acids (Gibco, 11,140), and 1 mM sodium pyruvate (Gibco, 11,360).

### Reagents and antibodies

Doxorubicin (c-200,923) was purchased from Santa Cruz Biotechnology. 3-methyladenine (M9281) was purchased from Sigma-Aldrich. Primary antibodies against histone H3 (4499), lamin A/C (4777), SQSTM1/P62 (5114), SUMO-1 (4930) and p-histone H2A.X (Ser139, 9718) were purchased from Cell Signaling Technology. GAPDH (sc-25,778) and UBC9 (sc-10,759) were purchased from Santa Cruz Biotechnology. LC3B (L7543) was purchased from Sigma Aldrich. Peroxidase-labeled antibody to mouse IgG (074–1802) and peroxidase-labeled antibody to rabbit IgG (074–1516) were purchased from Kirkegaard and Perry Laboratories. Alexa Fluor 488 goat anti-rabbit (A21206) and Alexa Fluor 647 donkey anti-mouse (A21235) were purchased from Molecular Probes.

### Plasmids and RNA interference

mRFP-LC3 (21075) and LAMP1-mGFP (34831) were obtained from Addgene. EGFP-LC3 and *ATG7*-siRNA plasmids were constructed by GeneChem Co. LTD (Shanghai, China). Plasmids were transfected into MDA-MB-231 and MCF-7 cells using Lipofectamine 3000 transfection reagent (Invitrogen, L3000). The transfection mixture was removed and replaced with fresh complete medium after 24 h incubation. The sequences of UBC9 shRNA were 5′-TTGGCAGTAAATCGTGTAGGCC-3′ (sh-UBC9–1#) and 5′-ATTTAGAAGTTCCTGTATTCCT-3′ (sh-UBC9–3#).

293FT cells were transfected with pLP1, pLP2, pLP/VSVG (Invitrogen, K4975) and sh-UBC9 or sh-Con plasmid. Supernatants containing the lentivirus were harvested after 48 h incubation and used to infect target cells. MDA-MB-231 and MCF-7 cells were selected with puromycin (4–8 μg/mL) (Sigma, P9620) to establish stable cell lines.

### Preparation of nuclear and cytosolic fractions

Cells were harvested by centrifugation at 1800 g for 10 min at 4 °C. After being washed twice with ice-cold PBS, cell pellets were suspended in five volumes of ice-cold buffer A (20 mM HEPES, 10 mM KCl, 1.5 mM MgCl_2_, 1 mM EDTA, 1 mM EGTA, 1 mM Na_3_VO_4_, 2 mM leupeptin, 1 mM PMSF, 1 mM DTT, 2 mM pepstatin A, and 250 mM sucrose). After sitting on ice for 15 min, the cells were homogenized by passing them through a 22-gauge needle 25 times. The nuclei were centrifuged at 1000 g for 10 min at 4 °C. The supernatant was carefully removed from the nuclear pellet and subjected to a further centrifugation at 100,000 g for 30 min, and this supernatant was considered the cytosolic fraction.

### Western blots and immunoprecipitation

Cells were lysed in RIPA lysis buffer (P0013B, Beyotime Biotechnology, Shanghai, China) supplemented with 10 mM NEM (N-ethylmaleimide; Sigma, E3876) to obtain total protein lysates. Nuclei were lysed in RIPA lysis buffer supplemented with 10 mM NEM to obtain nuclear lysates. Protein concentrations were measured using an enhanced BCA protein assay kit (P0009, Beyotime Biotechnology, Shanghai, China). The protein lysates were separated by SDS-PAGE, and proteins transferred to PVDF membranes. Membranes were blocked with 5% fat-free dry milk in Tris-buffered saline (10 mM Tris-base, 150 mM NaCl, pH 7.6) supplemented with 0.1% Tween 20 and incubated with primary antibodies at 4 °C overnight. Protein bands were detected by incubation with horseradish peroxidase-conjugated antibodies (Kirkegaard and Perry Laboratories) and visualized with Clarity Western ECL Substrate (Bio-Rad). For immunoprecipitation, total or nuclear protein lysates were obtained as described and equal quantities of proteins were incubated with primary antibodies at 4 °C on a rocking platform overnight. Immune complexes were collected with protein A/G agarose beads (Beyotime Technology), washed five times with phosphate-buffered saline (PBS), and subjected to SDS-PAGE and western blot. Quantification relative to.

GAPDH/Histone3 by densitometric analysis using the Quality One software (Bio-Rad).

### Confocal microscopy

Cells were seeded on coverslips and cultured in 24-well plates at 37 °C. After incubation, cells were fixed with 4% formaldehyde (Beyotime Biotechnology, P0098) for 30 min, permeabilized with 0.1% Triton X-100 (Amresco, 0694) in PBS for 10 min and blocked with 10% goat serum (Beyotime Biotechnology, C0265) in PBS for 30 min. Cells were incubated with primary antibodies at 4 °C overnight, then with the appropriate secondary antibodies at room temperature. Images were captured using a laser-scanning confocal microscope (Zeiss, Germany).

### Statistical analysis

Results are expressed as mean ± SD. The comparisons were performed using one-way analysis of variance (ANOVA). *P < 0.05 and **P < 0.01 were regarded as significant.

## Results

### Induction of nuclear DNA leakage activates nucleophagy

**Fig. 1:**
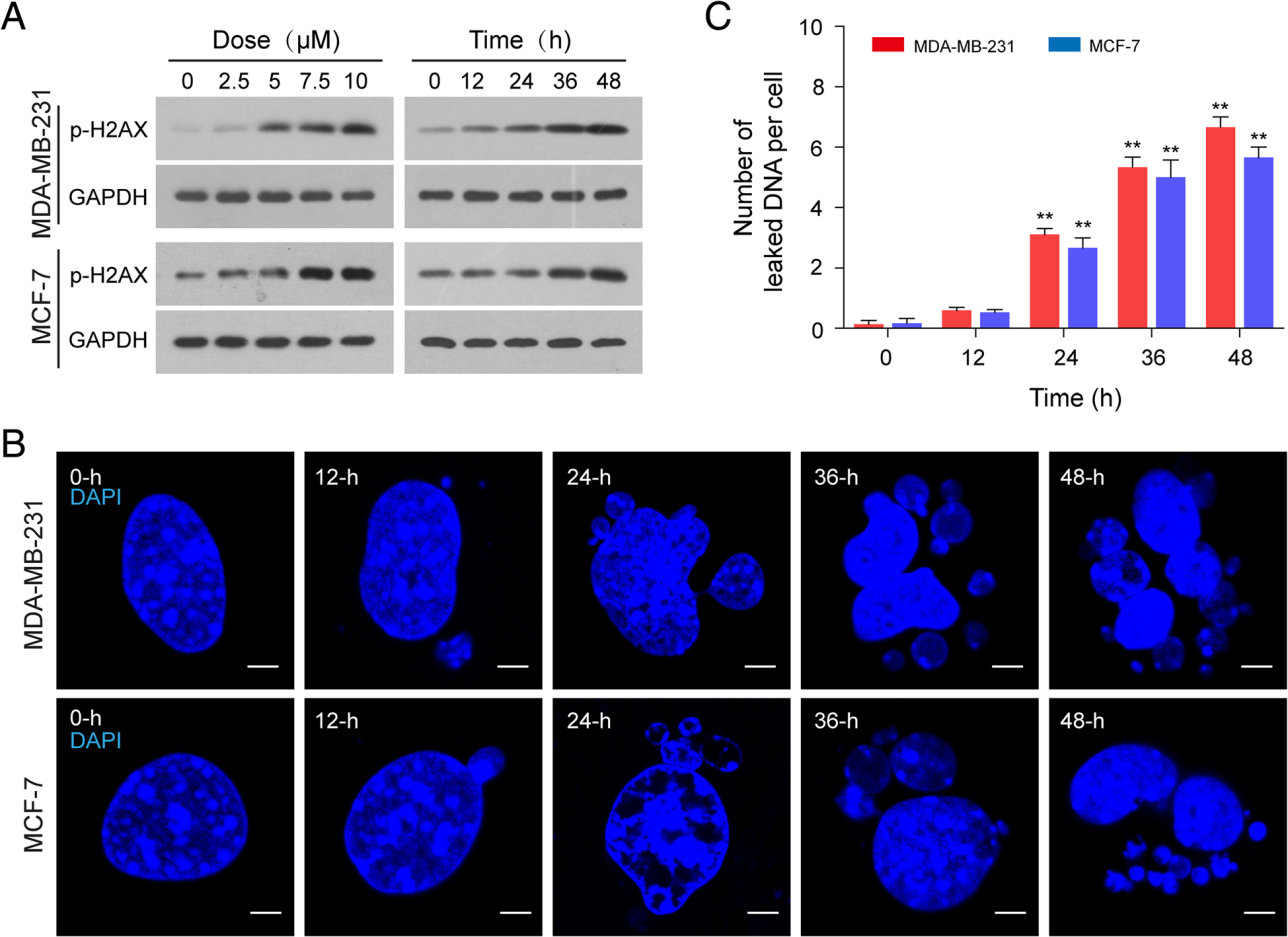
Doxorubicin (DOX) induces DNA damage and nuclear DNA leakage. Human breast cancer MDA-MB-231 and MCF-7 cells were treated without or with DOX as indicated. (**a**) The expression of phospho-H2AX (Ser139) was detected by western blot analysis with GAPDH as loading control. (**b**) Nuclear DNA leakage was shown by fluorescence microscopy with DAPI staining. Scale bars: 5 μm. (**c**) The number of DAPI-stained nuclear DNA particles leaked per cell was quantified from 50 cells in three independent experiments. Data was presented as mean ± S.D. (**P < 0.01)

Since doxorubicin (DOX) is known to cause DNA damage, we first examined the effects of doxorubicin on phosphorylation of H2AX (Ser139), a key DNA damage signal, in human breast cancer cells, MDA-MB-231 and MCF-7. As shown in Fig. 1a, treatment of human breast cancer cells with DOX increased the level of phosphor-H2AX (Ser139) in a dose- and time-dependent manner. Increasing evidence has revealed that nuclear DNA leakage often occurs in tumor cells with compromised DNA damage [26]. We next visualized DNA fragments generated by DNA damage using advanced 4D fluorescence microscopy. As shown in Fig. 1b and c, treatment of MDA-MB-231 and MCF-7 cells with the DNA damage agent, DOX, 10 μM, for 12 h resulted in modest increases in nuclear DNA leakage as identified by DAPI-positive particles outside the nucleus. These events became more apparent after 24 h of DOX treatment, and reached near-maximal levels after 36 h and 48 h of treatment.

**Fig. 2:**
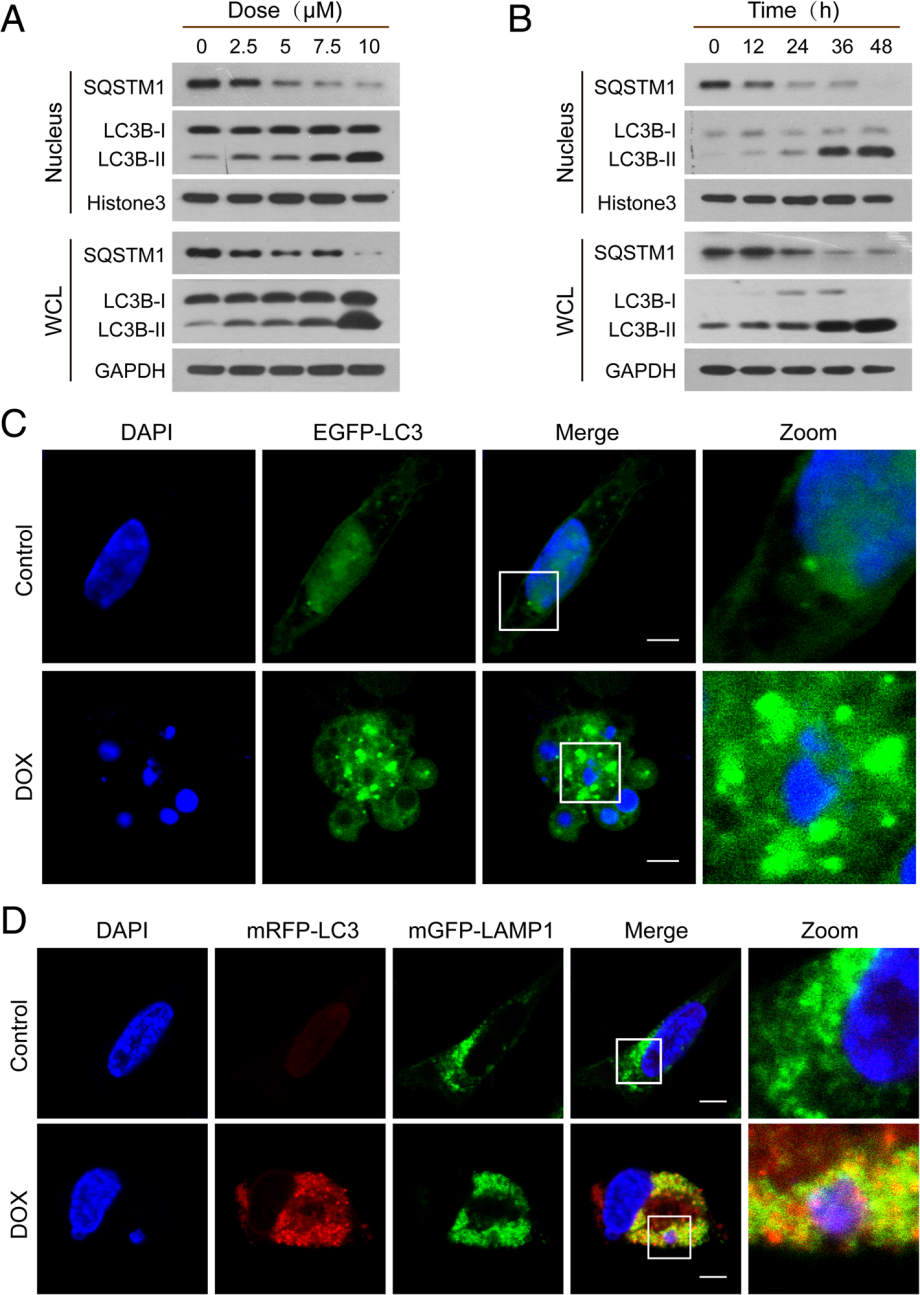
Nuclear DNA leakage activates nucleophagy**.** (**a** and **b**) MDA-MB-231 cells were exposed to various concentrations of DOX for 24 h, or treated with DOX (10 μM) for different times. The expressions of autophagy-related proteins, LC3B-I/LC3B-II and SQSTM1, in whole cellular (WCL) and nuclear extracts (nucleus) were detected by western blot. (**c**) MDA-MB-231 cells transfected with EGFP-LC3 were treated with DOX (10 μM) for 24 h. Cells were stained with DAPI and the colocalization of EGFP-LC3 (green) and leaked DNA (blue) was examined by confocal microscopy. Scale bars: 10 μm. (**d**) Confocal microscopy images of MDA-MB-231 cells treated without or with DOX (10 μM) for 24 h after co-expression of mRFP-LC3 (red) and mGFP-LAMP1 (green) and staining with DAPI (blue). Scale bars: 10 μm

It has been shown that after DNA damage, a selective form of autophagy known as nucleophagy can degrade nuclear components, thereby contributing to the maintenance of nuclear function and integrity [27]. To investigate the potential contribution of autophagy to nuclear DNA leakage/DNA damage, we evaluated autophagy activity by examination of LC3B conversion in both whole-cell and nuclear extracts. Western blot analysis showed that treatment of MDA-MB-231 and MCF-7 cells with DOX resulted in a dose- and time-dependent accumulation of LC3B-II in either whole-cell or nuclear extracts (Fig. 2a and b, Additional file 1: Figure S1A and S1B). A decrease in the level of the ubiquitin-binding receptor protein, SQSTM1, was also observed in both whole-cell and nuclear extracts of these cell lines in a dose- and time-dependent manner (Fig. 2a and b, Additional file 1: Figure S1A and S1B). To further determine the characteristics of nucleophagy, MDA-MB-231 and MCF-7 cells were transiently transfected with EGFP-LC3 and stained with DAPI. Autophagosome accumulation was detected by using a confocal laser-scanning microscope. An increased colocalization of autophagosomes (EGFP-LC3, green) and leaked nuclear DNA (blue) was observed in cells treated with DOX compared to control cells (Fig. 2c, Additional file 1: Figure S1C). In the late stage of autophagy, fusion of autophagosomes with lysosomes leads to the formation of autolysosomes. To visualize the overlap of autophagosomes and lysosomes with nuclear leaked DNA, we transiently transfected cells with mRFP-LC3 (red) and mGFP-LAMP1 (green) to study their colocalization with nuclear leaked DNA (blue). In DOX-treated cells, the leaked nuclear DNA was colocalized with LC3 and LAMP1, likely reflecting autophagosome-lysosome fusion, leading leaked nuclear DNA into the lysosomal degradation pathway (Fig. 2d, Additional file 1: Figure S1D).

To determine whether DOX-mediated nucleophagy observed in breast cancer cells also occur in other cancer cell lines, parallel studies were carried out in HepG2 (human hepatocellular carcinoma cell line), DU145 (human prostate cancer cell line), and A549 (human lung adenocarcinoma cell line) cells. Similarly, DOX treatment caused nucleophagy in these cells (Additional file 1: Figure S2A, S2B and S2C). Together, these results suggest that DNA damage induces leakage of nuclear DNA, leading to nucleophagy.

### LC3-lamin A/C interaction is required for nucleophagy

**Fig. 3:**
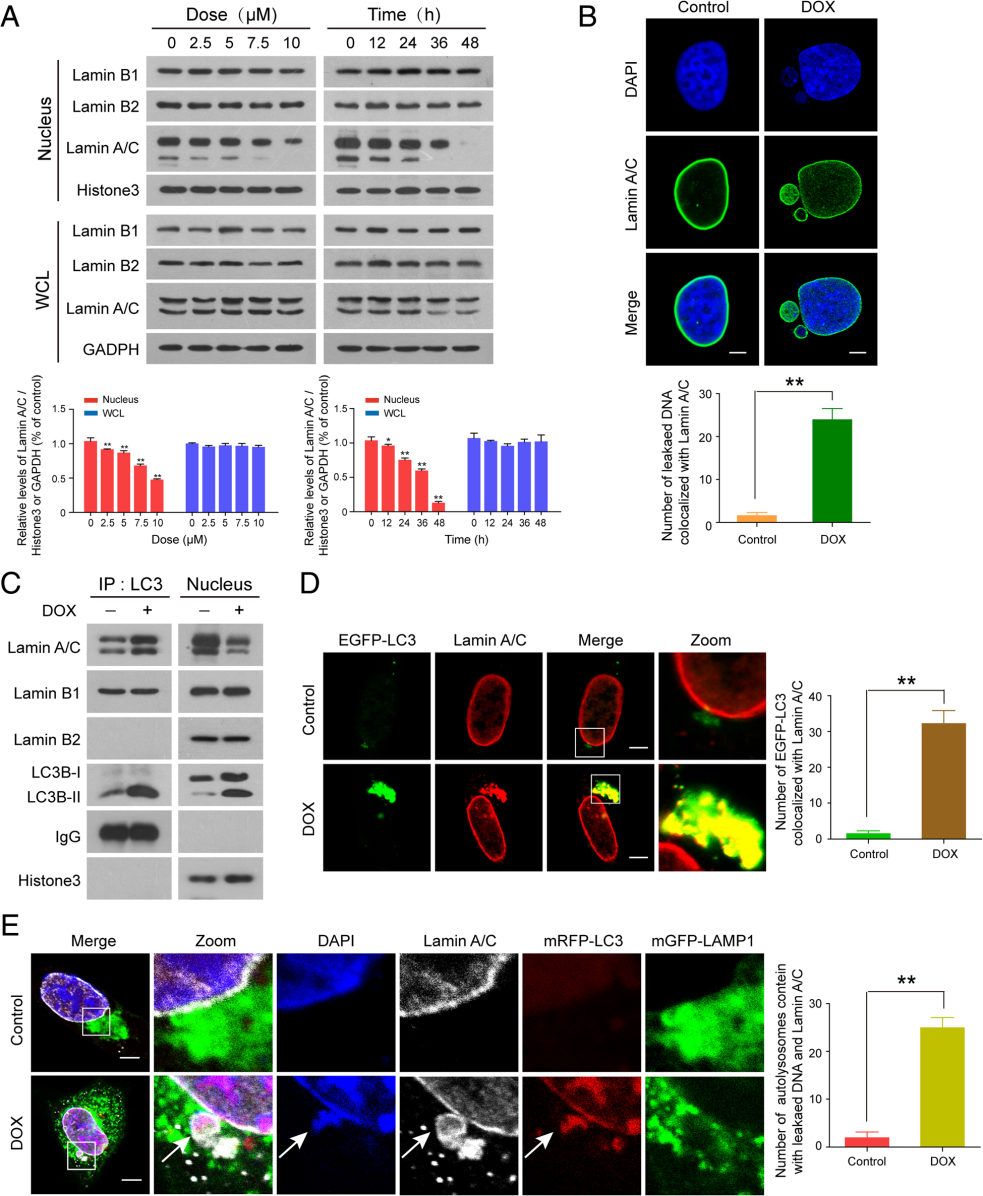
LC3-lamin A/C interaction is required for nucleophagy. MDA-MB-231 cells were treated without or with DOX as indicated. (**a**) The expressions of nuclear proteins, lamin A/C, lamin B1, and lamin B2, in whole cellular (WCL) and nuclear extracts (nucleus) were detected by western blot. Comparison of the intensities were statistically estimated and represented as mean ± SD for three independent experiments (*P < 0.05, **P < 0.01). (**b**) The colocalization of lamin A/C (green) and leaked nuclear DNA (blue) was determined by confocal microscopy. Scale bars: 10 μm. The number of DAPI-stained particles with leaked nuclear DNA colocalized with lamin A/C was quantified from 30 cells in three independent experiments. Data was presented as mean ± S.D. (**P < 0.01). (**c**) After treatment with DOX (10 μM, 24 h), nuclear lysates were prepared and subjected to immunoprecipitation using anti-LC3, and the associated LC3B-I/LC3B-II, lamin A/C, lamin B1, and lamin B2 were determined by immunoblotting. (**d**) MDA-MB-231 cells were transfected with EGFP-LC3, treated as in (**b**), and examined by confocal microscopy to determine the colocalization of EGFP-LC3 and lamin A/C (red). Scale bars: 10 μm. The number of EGFP-LC3 colocalized with lamin A/C was quantified from 30 cells in three independent experiments. Data was presented as mean ± S.D. (**P < 0.01). (**e**) Immunofluorescence analysis showed the colocalization of leaked DNA (blue) with mRFP-LC3(red), mGFP-LAMP1(green), and lamin A/C (white) in MDA-MB-231 cells treated with DOX (10 μM, 24 h). Scale bars: 10 μm. The number of autolysosomes containing leaked DNA and lamin A/C was quantified from 50 cells in three independent experiments. Data was presented as mean ± S.D. (**P < 0.01)

Since nuclear lamins have an important role in the maintenance of nuclear architecture, we examined whether levels of lamins were altered during DNA damage. Western blot analysis showed that treatment of human breast cancer MDA-MB-231 and MCF-7 cells with doxorubicin decreased the levels of lamin A/C in the nucleus in a dose- and time-dependent manner, but had little effect on the expression of lamin A/C in whole cellular extracts, suggesting that DNA damage may promote degradation of lamin A/C. In contrast, the expression of lamin B1 and lamin B2 in either nucleus or whole cellular extract was not altered in response to DNA damage (Fig. 3a, Additional file 1: Figure S3A). To determine whether cleavage of lamin A/C contributes to nuclear DNA leakage, immunofluorescence and confocal microscopy were employed. As shown in Fig. 3b and Additional file 1: Figure S3B, the nuclei of cells treated without or with DOX were surrounded peripherally by a continuous lamin A/C network (green signals). Interestingly, the leaked nuclear DNA was not only surrounded by lamin A/C (green signals), but also colocalized with lamin A/C in cells treated with DOX, suggesting that lamin A/C might participate in nuclear DNA leakage.

If nuclear envelope proteins such as lamin A/C are targeted by nucleophagy, one would expect an interaction of these components with autophagy proteins. To test this possibility, we performed immunoprecipitation assays to determine the interaction of LC3 with lamin A/C and lamin B. As shown in Fig. 3c and Additional file 1: Figure S3C, the interaction of LC3 with lamin A/C was increased in nuclei of cells treated with DOX. In contrast, the interaction of LC3 with lamin B1 was not changed by DOX treatment, and the interaction of LC3 with lamin B2 was not observed in cells treated without or with DOX. Immunofluorescence microscopic studies revealed a colocalization of LC3 and lamin A/C after DNA damage (Fig. 3d and Additional file 1: Figure S3D). To determine whether LC3-lamin A/C interaction was required for nucleophagy, immunofluorescence analysis of lamin A/C (white), mRFP-LC3 (red), mGFP-LAMP1 (green), and DAPI (blue) was performed. We found that leaked nuclear DNA (DAPI signals) colocalized with mRFP-LC3, mGFP-LAMP1, and lamin A/C after DNA damage (Fig. 3e and Additional file 1: Figure S3E).

**Fig. 4:**
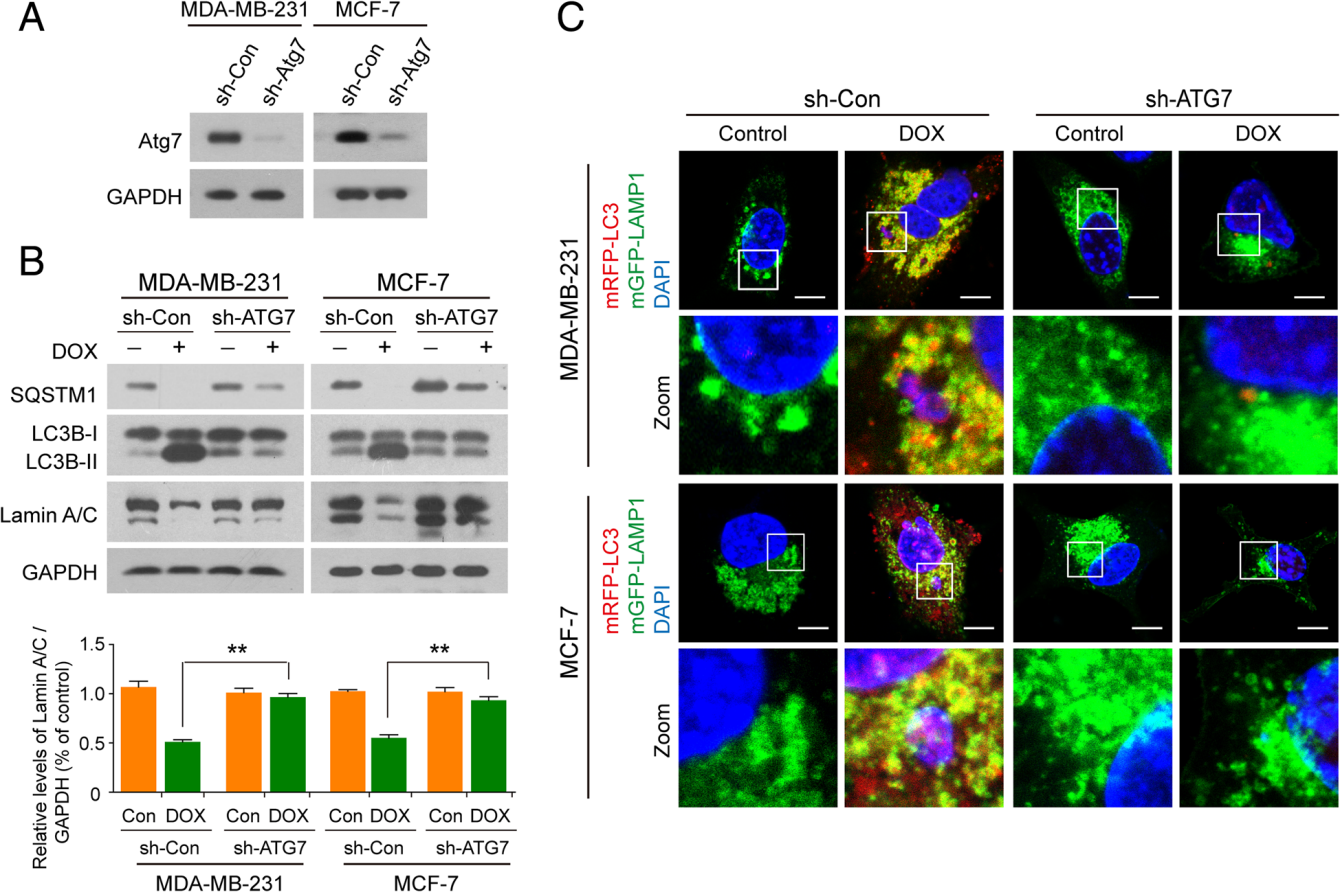
Inhibiting autophagy impairs degradation of lamin A/C. MDA-MB-231 and MCF7 cells stably expressing control shRNA (sh-Control) or ATG7-shRNA (sh-ATG7) were treated without or with DOX (10 μM) for 24 h. (**a**) The expression of ATG7 in MDA-MB-231 and MCF7 cells. (**b**) Nuclear extracts were prepared and subjected to western blot analysis using antibodies against LC3B, SQSTM1, and lamin A/C. Data was presented as mean ± S.D. (**P < 0.01). (**c**) The localization of mRFP-LC3 (red), mGFP-LAMP1 (green) and leaked DNA (blue) was evaluated by confocal microscopy. Scale bars: 10 μm

To ascertain whether nucleophagy contributed to the degradation of lamin A/C, we investigated the consequences of autophagy inhibition by incubating cells with 3-methyladenine (3-MA), a widely used autophagy inhibitor. Treating cells with 3-MA markedly inhibited the formation of nucleophagosomes as indicated in reduction of LC3B-II expression in nuclei and noticeably attenuated DOX-induced nucleophagosome-lysosome fusion (Additional file 1: Figure S4A and S4B). Inhibition of nucleophagy by 3-MA also effectively impaired the downregulation of lamin A/C in nuclei mediated by DNA damage (Additional file 1: Figure S4A). To exclude the possibility of nonspecific effects of 3-MA, a siRNA approach was used to stably knock down autophagy-related protein 7 (*ATG7*) expression (Fig. 4a). Knockdown of *ATG7* reduced DOX-mediated LC3B-II accumulation in nuclei and nucleophagosome-lysosome fusion (Fig. 4b and c). Knockdown of *ATG7* also markedly attenuated DOX-mediated downregulation of lamin A/C in nuclei (Fig. 4b). Taken together, these results indicate that LC3-lamin A/C interaction is required for nucleophagy, which results in degradation of nuclear components like lamin A/C and leaked nuclear DNA.

### SUMOylation of lamin A/C is required for LC3-lamin A/C interaction

**Fig. 5:**
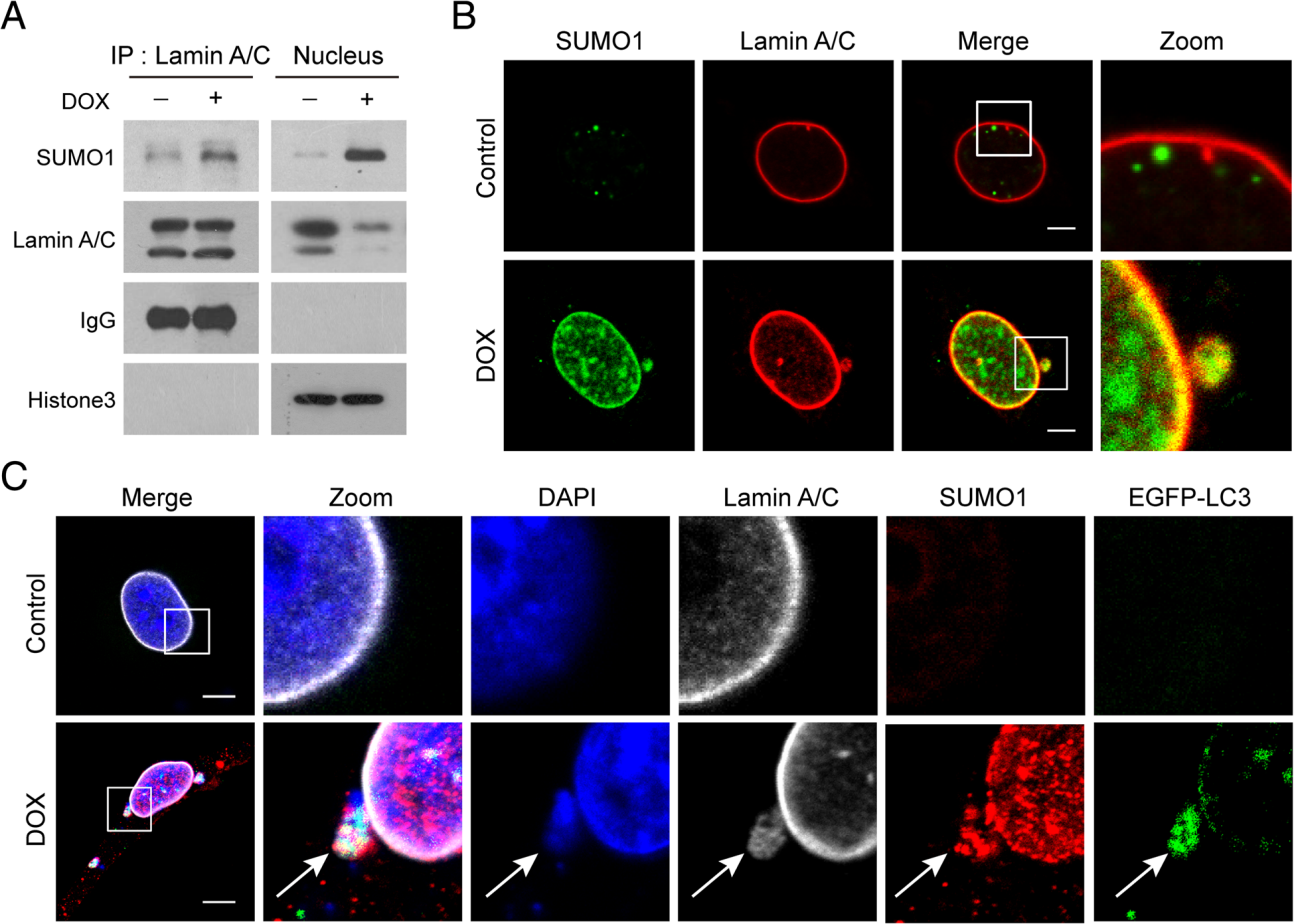
Lamin A/C is SUMOylated in response to DNA damage. MDA-MB-231 cells were treated without or with DOX (10 μM) for 24 h. (**a**) Nuclear extracts were prepared and immunoprecipitated using anti-lamin A/C, and immunoblotted using anti-SUMO1. (**b**) Immunofluorescence analysis revealed colocalization of lamin A/C (red) and SUMO1 (green). Scale bars: 10 μm. (**c**) Immunofluorescence analysis showed a colocalization of SUMO1 (red) with EGFP-LC3 (green), lamin A/C (white) and leaked nuclear DNA (blue). Scale bars: 10 μm

A few studies have implicated SUMOylation in the regulation of A-type lamins (lamin A/C) [24, 28]. Because lamin A/C protein exhibits a characteristic pattern of localization at the nuclear periphery [29], we hypothesized that SUMOylation of lamin A/C may be important for this localization pattern. To test this, we immunoprecipitated SUMO1 from nuclear extracts of cells treated without or with DOX, immunoblotted using anti-lamin A/C, and found that SUMO1 was precipitated with lamin A/C in response to DNA damage (Fig. 5a and Additional file 1: Figure S5A). Furthermore, immunofluorescence analysis revealed a colocalization of lamin A/C (red) and SUMO1 (green) (Fig. 5b and Additional file 1: Figure S5B). To investigate whether SUMOylation of lamin A/C was involved in LC3-lamin A/C interaction which contributes to occurrence of nucleophagy, immunofluorescence analysis was employed. We found that SUMO1 colocalized with LC3, lamin A/C and leaked nuclear DNA in response to DNA damage (Fig. 5c and Additional file 1: Figure S5C). These results suggest that SUMOylation of lamin A/C is required for LC3-lamin A/C interaction in response to DNA damage.

### Nuclear accumulation of UBC9 contributes to SUMOylation of lamin A/C and nucleophagy

**Fig. 6:**
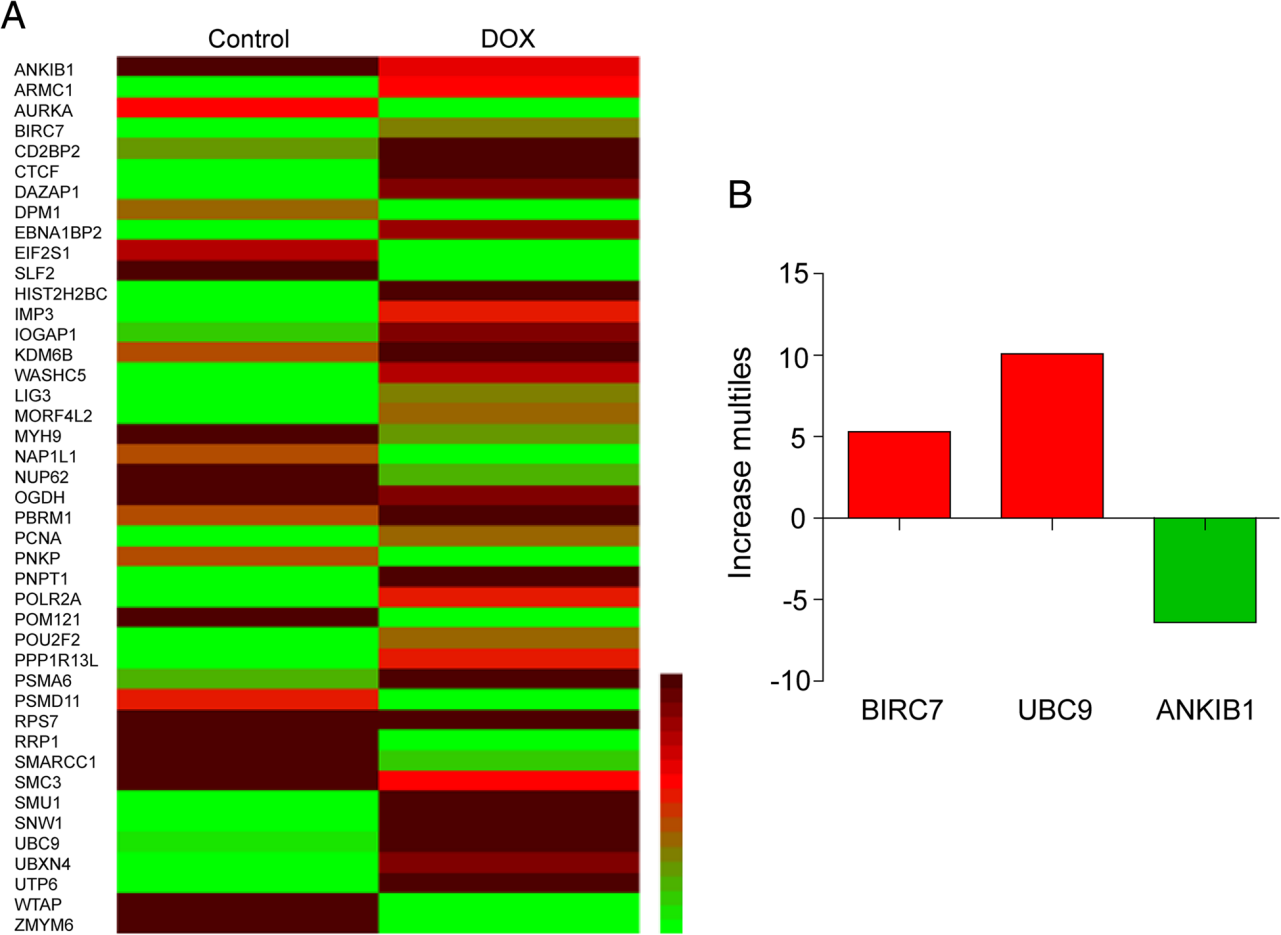
Proteomic assay identifies UBC9 as enzyme responsible for SUMOylation of lamin A/C. MDA-MB-231 cells were treated with DOX (10 μM) for 24 h, nuclear extracts were prepared and subjected to proteomic analysis using LC-ESI-Q-TOF MS/MS. (**a**) Heatmap showing differential protein expression. (**b**) Only three proteins were related to post-translation modification

A substantial body of evidence demonstrates that SUMOylation is catalyzed by an E1-activating enzyme (SAE1/2), an E2-conjugating enzyme (UBC9) and multiple E3 ligases. To identify which enzyme was responsible for the SUMOylation of lamin A/C, a proteomic approach was employed. We treated MDA-MB-231 cells without or with DOX for 24 h, separated nuclear proteins by 1D-SDS–polyacrylamide gel electrophoresis, and then identified peptides from the gel slices by liquid chromatography-mass spectrometry (LC-ESI-Q-TOF MS/MS). We found 43 proteins that were upregulated or downregulated at least 2-fold (Fig. 6a). Among these proteins, BIRC7 and UBC9 (upregulated) and ANKIB1 (downregulated) are related to enzymes that play important roles in the regulation of post-translational protein modification, and only UBC9 contributes to SUMOylation (Fig. 6b).

**Fig. 7:**
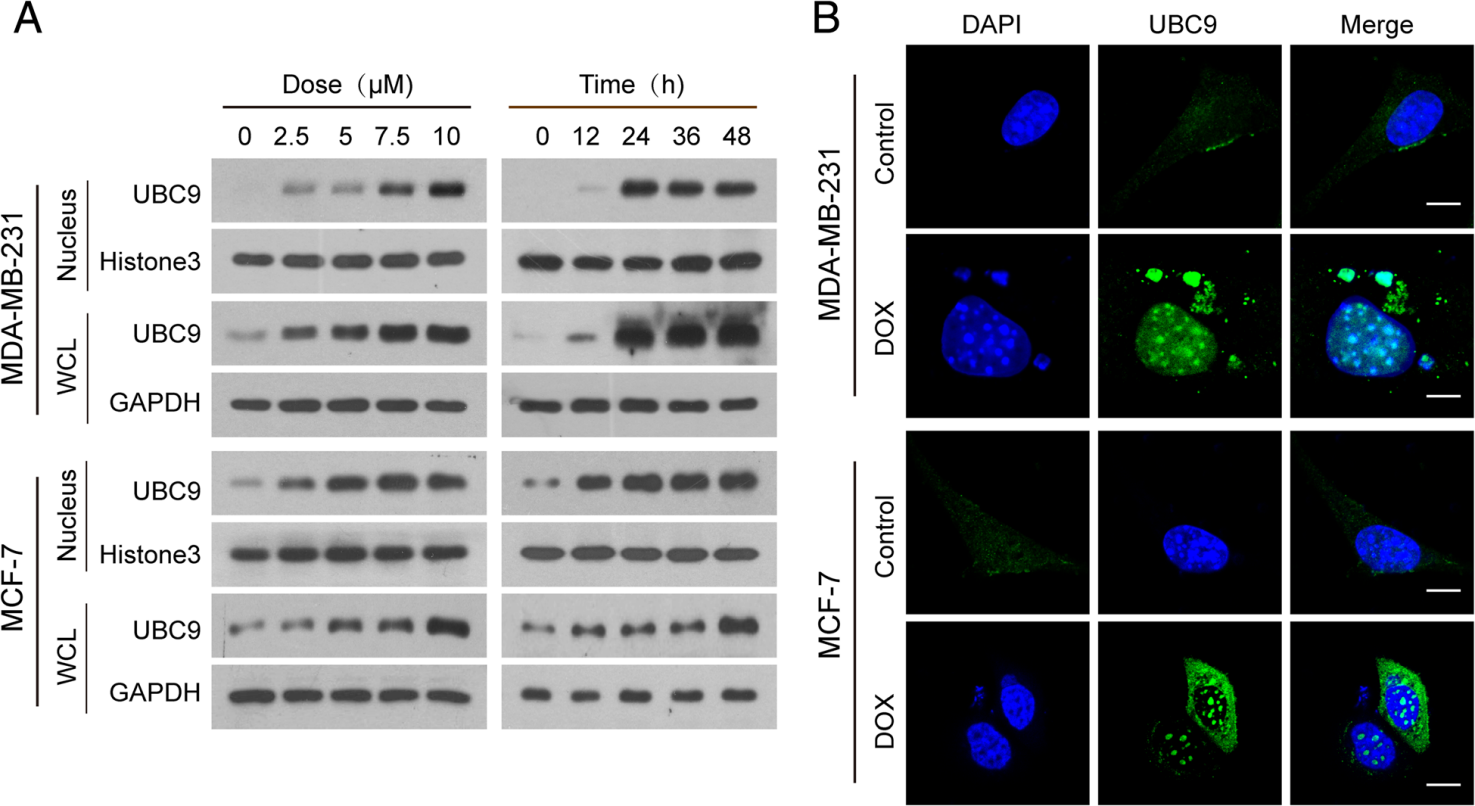
UBC9 accumulates in nucleus in response to DNA damage. MDA-MB-231 and MCF7 cells were treated with DOX (10 μM) for 24 h. (**a**) The expression of UBC9 in whole cellular (WCL) and nuclear extracts (nucleus) was detected by western blot analysis. (**b**) Confocal microscopy images showed accumulation of UBC9 in nucleus. Scale bars: 10 μm

Because SUMOylation mainly occurs in the nucleus, we hypothesized that UBC9 may be accumulating in the nucleus in response to DNA damage. To test this, we examined the expression of UBC9 in whole cellular and nuclear extracts of cells treated with DOX. Treatment of human breast cancer MDA-MB-231 and MCF-7 cells with DOX resulted in significant increases in UBC9 in both whole cellular and nuclear extracts in a dose- and time-dependent manner (Fig. 7a). Immunofluorescence analysis revealed that increases in localization of UBC9 in the nuclei (including leaked nuclei) were observed in cells treated with DOX (Fig. 7b). It had been shown previously that the ubiquitin-conjugating enzyme UBC9, which conjugates SUMO1 to target proteins, interacted with lamin A/C [23–25]. We hypothesized that UBC9 contributed to SUMOylation of lamin A/C through interaction with lamin A/C. To test this, we immunoprecipitated UBC9 from nuclear extracts of cells treated without or with DOX and immunoblotted using anti-SUMO1 and anti-lamin A/C and found that UBC9 co-immunoprecipitated with SUMO1 and lamin A/C in nuclear extracts of cells treated with DOX (Additional file 1: Figure S6A). Furthermore, immunofluorescence analysis revealed that UBC9 was colocalized with lamin A/C in nuclei (including leaked nuclei) of cells in response to DNA damage (Additional file 1: Figure S6B). Taken together, these data indicate that accumulation of UBC9 in nucleus and its interaction with lamin A/C are required for SUMOylation of lamin A/C in response to DNA damage.

### Knockdown of UBC9 attenuates SUMOylation of lamin A/C and nucleophagy mediated by DNA damage

**Fig. 8:**
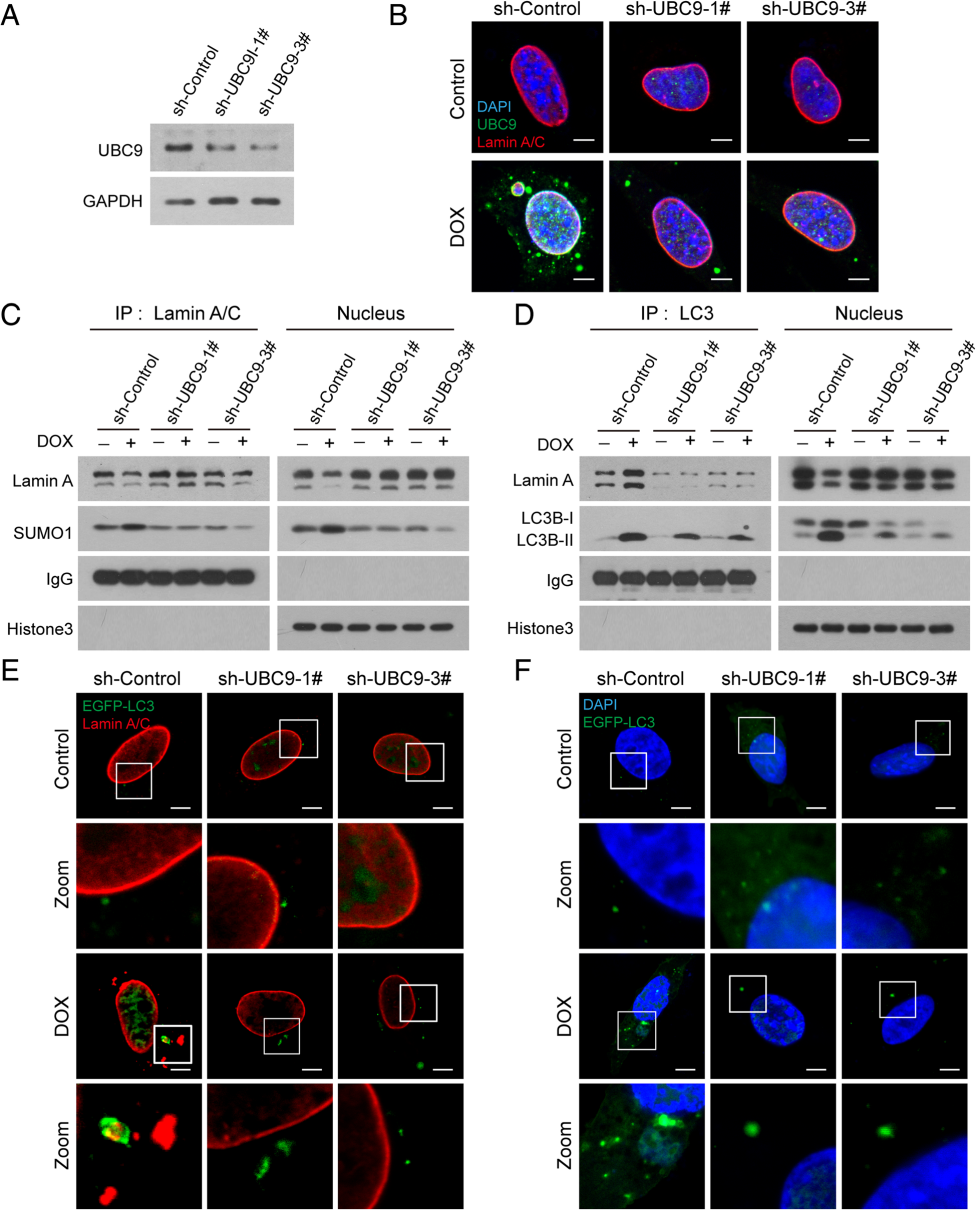
Knockdown of UBC9 attenuates SUMOylation of lamin A/C and nucleophagy mediated by DNA damage. MDA-MB-231 cells stably expressing sh-Control and sh-UBC9 (1# and 3#) were treated with DOX (10 μM) for 24 h. (**a**) The expression of UBC9 in sh-Control and sh-UBC9 cells was detected by western blot. (**b**) Nuclear extracts were prepared, immunoprecipitated using anti-lamin A/C, and immunoblotted using anti-SUMO1. (**c**) The colocalization of lamin A/C (red), SUMO1 (green) and leaked DNA (blue) was determined by immunofluorescence. Scale bars: 10 μm. (**d**) Nuclear extracts were prepared, immunoprecipitated with anti-LC3, and immunoblotted with anti-lamin A/C. (**e**) The colocalization of EGFP-LC3 (green) and lamin A/C (red) was detected by immunofluorescence. Scale bars: 10 μm. (**f**) The colocalization of EGFP-LC3 (green) and leaked DNA (blue) was detected by immunofluorescence. Scale bars: 10 μm

To further explore the functional role of UBC9 in regulating SUMOylation of lamin A/C and nucleophagy, two lentiviruses carrying shRNA (shUBC9–1# and shUBC9–3#) were employed to stably knock down UBC9 expression in MDA-MB-231 and MCF-7 cells (Fig. 8a and Additional file 1: Figure S7A). Immunopreciptation and western blot analyses showed that knockdown of UBC9 inhibited SUMOylation and degradation of lamin A/C mediated by DNA damage (Fig. 8b and c, and Additional file 1: Figure S7B and S7C). Knockdown of UBC9 also attenuated the interaction and colocalization of lamin A/C with LC3 and accumulation of LC3B-II in nuclei caused by DNA damage (Fig. 8d and e, and Additional file 1: Figure S7D and S7E). Finally, knockdown of UBC9 inhibited nucleophagy induced by DNA damage (Fig. 8f and Additional file 1: Figure S7F). These findings further confirm the important functional role of UBC9 in regulating SUMOylation of lamin A/C and nucleophagy.

## Discussion

In this study, we showed that lamin A/C was a selective nuclear autophagy substrate in response to DNA damage. We revealed that the autophagy protein LC3 interacted with the nuclear lamina protein lamin A/C. Through this interaction, nuclear autophagy specifically mediated degradation of lamina and leaked nuclear DNA. The autophagy protein LC3 played an essential role in the mechanism of autophagy [30, 31]. The LC3 protein is a key component of the autophagy pathway, contributing to both cargo selection and autophagosome formation in the cytoplasm [32, 33]. Increasing evidence has revealed that the nucleus also contains LC3, which plays a significant role in degradation of damaged nuclei and nuclear components [11, 30]. However, little is known about the mechanism of autophagy in degrading nuclear components. The degradation of nuclear components by the autophagy pathway is known as nucleophagy [7, 34], and increasing evidence has revealed that nuclear lamin proteins are involved in nucleophagy in mammalian cells. Dou Z et al. reported that the autophagy protein LC3/Atg8 directly interacted with the nuclear lamina protein, LMNB1 (lamin B1), and was bound to LMN/lamin-associated chromatin domains (LADs). Through these interactions, autophagy specifically mediated destruction of nuclear lamina during tumorigenic stress, such as oncogene activation or DNA damage [11]. Another report by Park YE et al. revealed that A-type lamins (lamin A/C) was involved in nucleophagy [8] by demonstrating that mutations in the genes encoding A-type lamins (*LMNA*) increased LC3-II protein levels, leading to degradation of nuclear components by nucleophagy. A recent study showed that arginine starvation reduced lamin A/C and induced nuclear DNA leakage in response to DNA damage, suggesting that a lack of lamin A/C structure can trigger autophagy-mediated degradation of damaged nuclear DNA [26]. In this study, we revealed that lamin A/C was a nucleophagy substrate in response to DNA damage, and demonstrated that LC3 interacted with lamin A/C, leading to degradation of lamin A/C and leaked nuclear DNA by nucleophagy. Firstly, increased expression of LC3B-II and decreased expression of SQSTM1 in the nucleus in response to DNA damage. Secondly, lamin A/C (but not lamin B1 or B2) was dramatically downregulated during DOX-induced DNA damage. Thirdly, by using immunoprecipitation analysis, we found that after DNA damage, LC3 interacted with lamin A/C in the nucleus, but not with lamin B1 and B2, while immunofluorescence data revealed that LC3 colocalized with lamin A/C, LAMP1, and leaked nuclear DNA. Finally, inhibition of autophagy by incubation with 3-MA or knocking down *ATG7* impaired LC3-lamin A/C interaction and lamin A/C degradation mediated by DNA damage. Altogether, these findings suggest that LC3-lamin A/C interaction may promote nucleophagy which mediates degradation of lamin A/C and leaked nuclear DNA.

We also demonstrated in this study, that lamin A/C was SUMOylated by the SUMO E2 enzyme, UBC9, and that this was required for LC3-lamin A/C interaction. SUMOylation plays an important role in regulating the functional properties of target proteins in cells, many associated with the nucleus [22, 35, 36], and it has been shown that SUMOylation is important for lamin A/C function [20, 24, 29]. Simon DN et al. reported that SUMO1 might contribute to modification of lamin A/C [24]. A previous study identified an interaction between lamin A/C and UBC9, the SUMO E2 protein, suggesting that lamin A/C could be a target of SUMOylation mediated by UBC9 [23]. In support of this model, our data provide evidence that UBC9-mediated SUMOylation contributes to LC3-lamin A/C interaction and nucleophagy. Immunoprecipitation and immunofluorescence assays revealed that lamin A/C was SUMOylated by SUMO1. By using a proteomic analysis, we found that only UBC9, which was upregulated in response to DNA damage, was involved in the regulation of SUMOylation. We also found that upregulated UBC9, which conjugates SUMO1 to target protein, could interact with either SUMO1 or lamin A/C, suggesting that UBC9 interacts with lamin A/C. The functional role of UBC9 in mediating SUMOylation of lamin A/C and nucleophagy was further confirmed by knockdown of UBC9 with shRNA, which noticeably attenuated SUMOylation of lamin A/C mediated by DNA damage. Knockdown of UBC9 also attenuated the interaction between lamin A/C and LC3. UBC9 deficiency also markedly decreased nucleophagy in mediating degradation of leaked nuclear DNA and nuclear components including lamin A/C. Therefore, our findings suggest that DNA damage triggers accumulation of UBC9 in nucleus, leading to SUMOylation of lamin A/C that is required for lamin A/C-LC3 interaction and nucleophagy.

## Conclusions

**Fig. 9:**
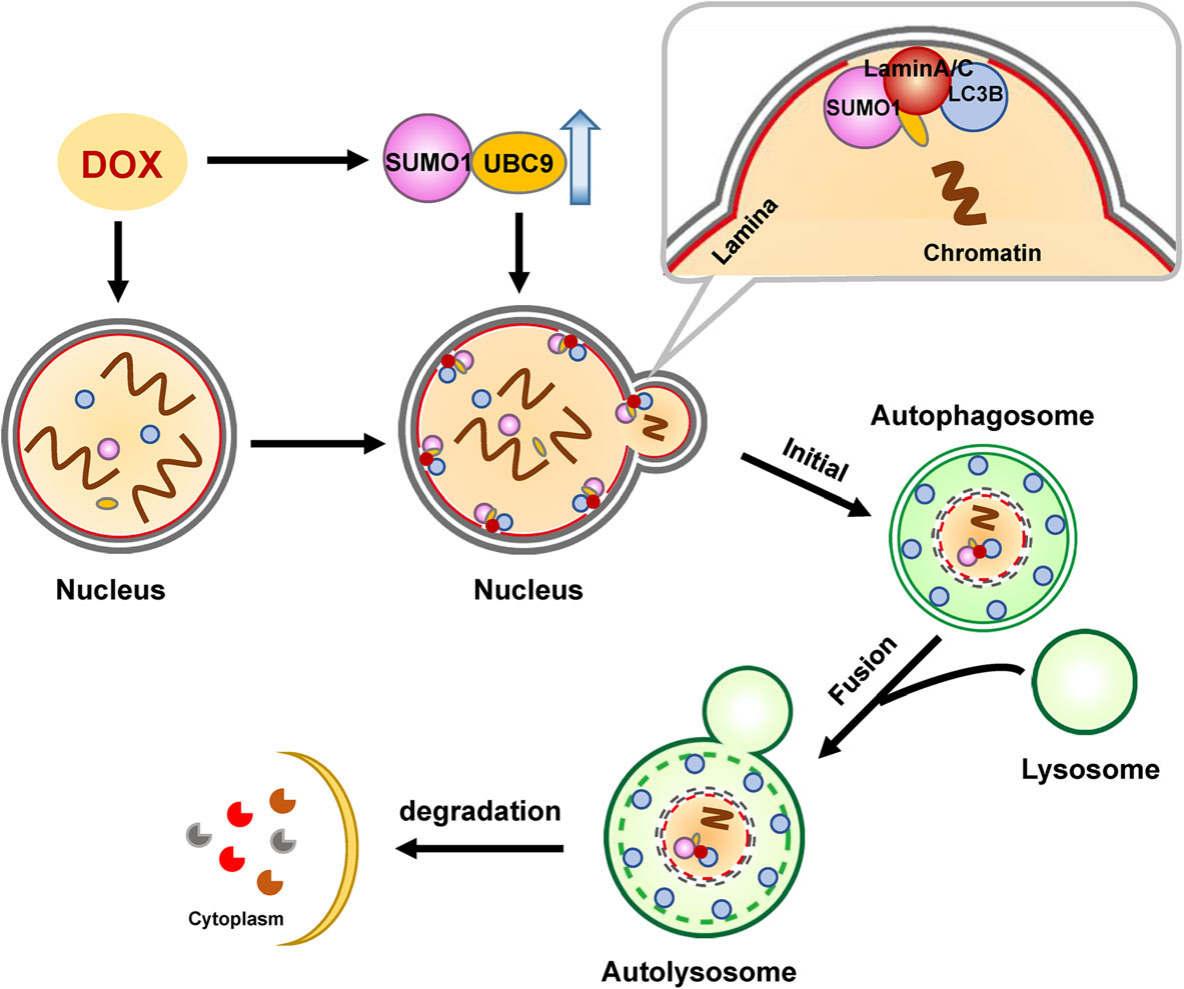
Autophagy-mediated degradation of lamin A/C and leaked nuclear DNA

These findings support a hypothetical model of DNA damage-activated nucleophagy (Fig. 9) in which DOX treatment induces DNA damage, leading to leakage of nuclear DNA into the cytoplasm. DNA damage also triggers nuclear accumulation of UBC9, leading to SUMOylation of lamin A/C, resulting in LC3-lamin A/C interaction, and culminating in the occurrence of nucleophagy, which degrades nuclear components including lamin A/C and leaked nuclear DNA.

## Additional file

Additional file 1: **Figure S1.** Nuclear DNA leakage activates nucleophagy. **Figure S2.** Nuclear autophagy exists in other cancer cell lines. **Figure S3.** LC3-lamin A/C interaction is required for nucleophagy. **Figure S4.** Inhibiting autophagy impairs degradation of lamin A/C. **Figure S5.** Lamin A/C is SUMOylated in response to DNA damage. **Figure S6.** UBC9 accumulates in nucleus in response to DNA damage. **Figure S7.** Knockdown of UBC9 attenuates SUMOylation of lamin A/C and nucleophagy mediated by DNA damage. (DOCX 6283 kb)

## Abbreviations

3-MA: 3-methyladenine
DAPI: 4′,6-diamidino-2-phenylindole
Dox: Doxorubicin
EGFP: Enhanced green fluorescent protein
LAMP: Lysosomal-associated membrane protein
LC3: Microtubule-associated protein 1 light chain 3
mRFP: Modified red fluorescent protein
shRNA: Short hairpin RNA
SQSTM1: Sequestosome 1
SUMOylation: Small ubiquitin-related modifier
UBC9: Ubiquitin conjugating enzyme E2I
