# Supplementary material for: Nuclear accumulation of UBC9 contributes to SUMOylation of lamin A/C and nucleophagy in response to DNA damage
Source: J Exp Clin Cancer Res. 2019 Feb 11;38:67. doi: 10.1186/s13046-019-1048-8 (PMC6371487; doi:10.1186/s13046-019-1048-8)
Supplement: Supplementary file 1 — Figure S1. Nuclear DNA leakage activates nucleophagy. Figure S2. Nuclear autophagy exists in other cancer cell lines. Figure S3. LC3-lamin A/C interaction is required for nucleophagy. Figure S4. Inhibiting autophagy impairs degradation of lamin A/C. Figure S5. Lamin A/C is SUMOylated in response to DNA damage. Figure S6. UBC9 accumulates in nucleus in response to DNA damage. Figure S7. Knockdown of UBC9 attenuates SUMOylation of lamin A/C and nucleophagy mediated by DNA damage. (DOCX 6283 kb) [file 13046_2019_1048_MOESM1_ESM.docx]

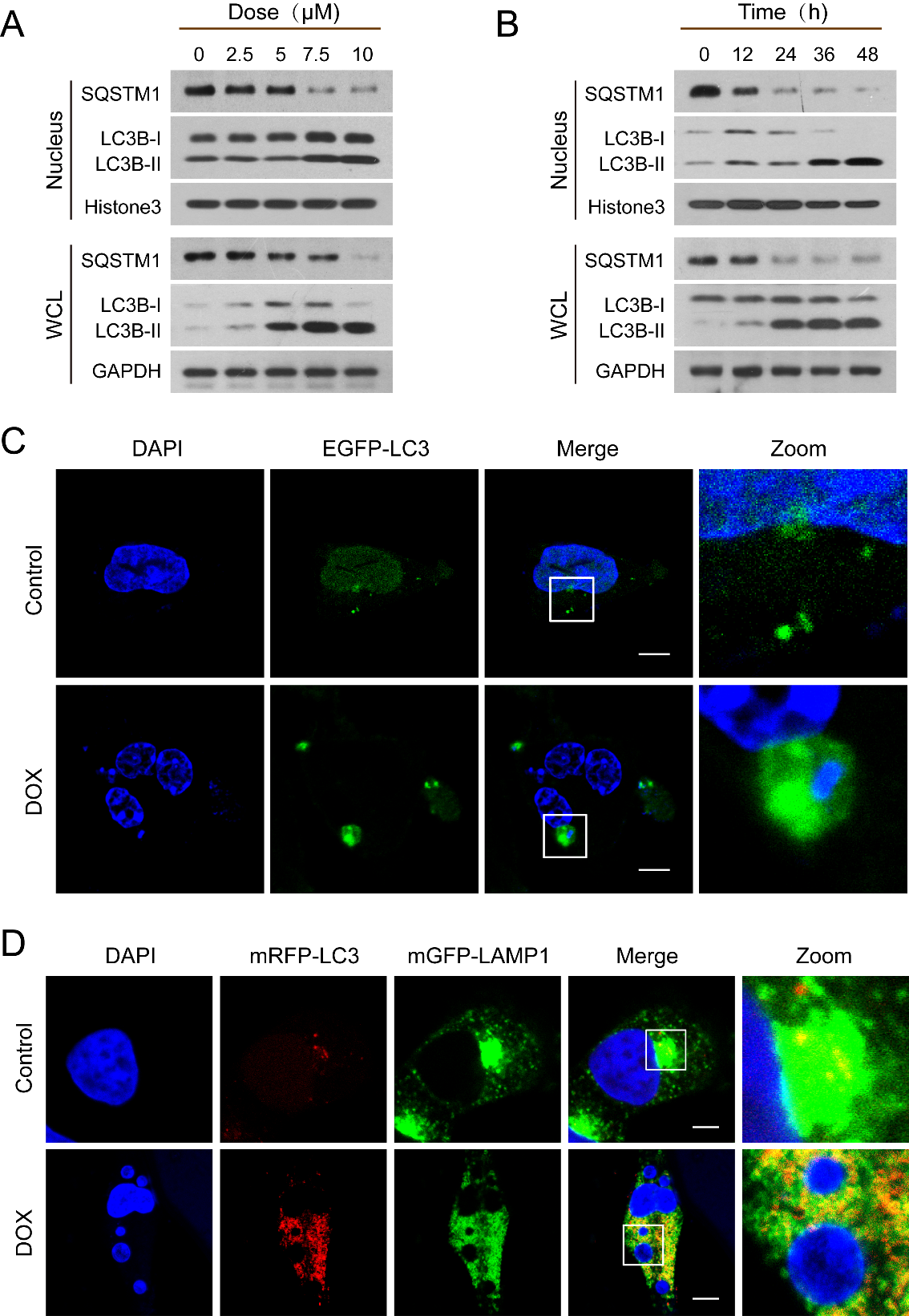


**Figure** **S1. Nuclear DNA leakage activates nucleophagy.** (A and B) MCF-7 cells were exposed to various concentrations of DOX for 24 h, or treated with DOX (10 μM) for different times. The expressions of autophagy-related proteins, LC3B-I/LC3B-II and SQSTM1, in whole cellular (WCL) and nuclear extracts (nucleus) were detected by western blot. (C) MCF-7 cells transfected with EGFP-LC3 were treated with DOX (10 μM) for 24 h. Cells were stained with DAPI and the colocalization of EGFP-LC3 (green) and leaked DNA (blue) was examined by confocal microscopy. Scale bars: 10 μm. (D) Confocal microscopy images of MCF-7 cells treated without or with DOX (10 μM) for 24 h after co-expression of mRFP-LC3 (red) and mGFP-LAMP1 (green) and staining with DAPI (blue). Scale bars: 10 μm.


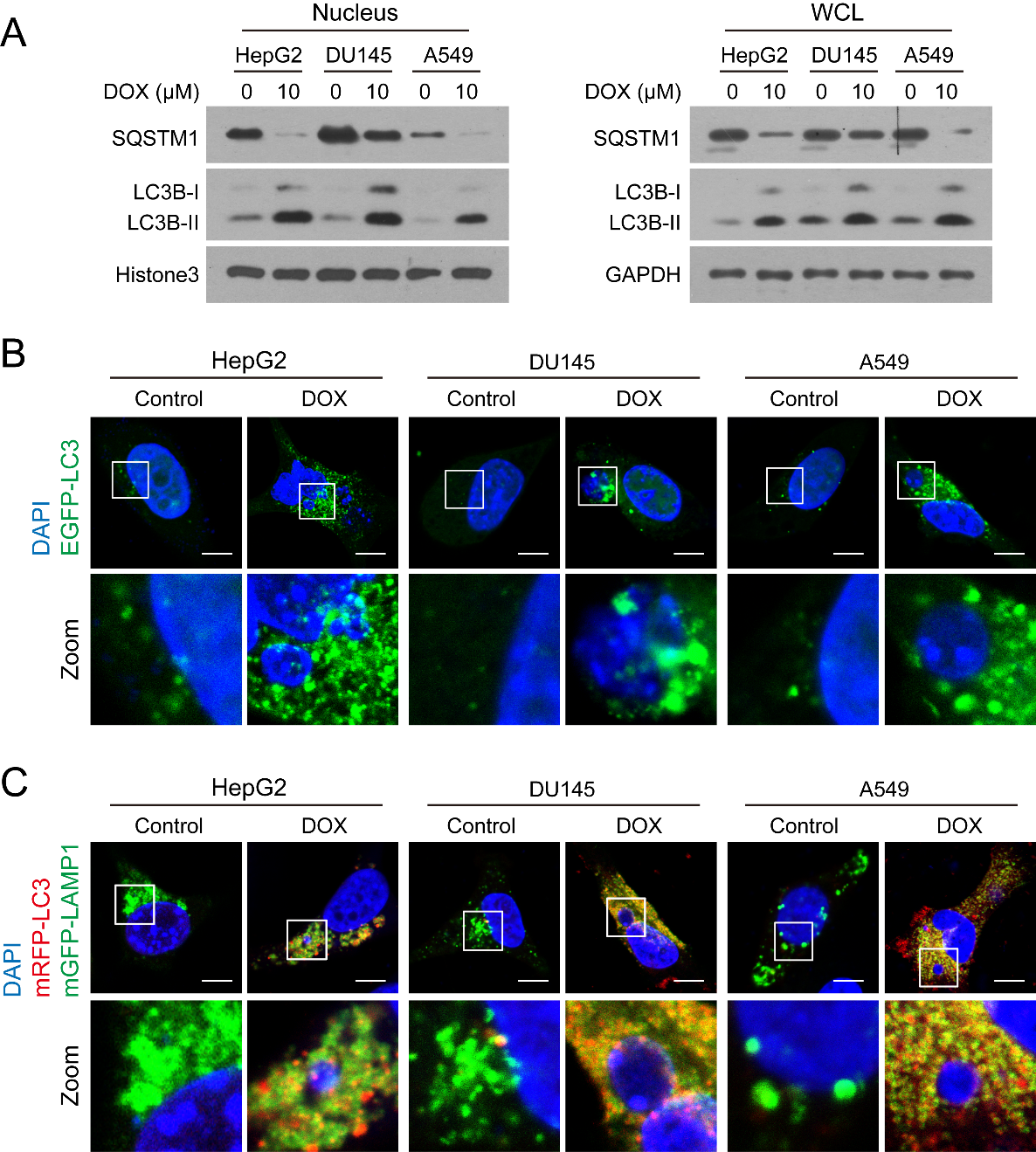


**Figure** **S2. Nuclear autophagy exists in other cancer cell lines.** (A) HepG2 (human hepatocellular carcinoma cell line), DU145 (human prostate cancer cell line), and A549 (human lung adenocarcinoma cell line) cells were exposed to DOX (10 μM) for 24 h. The expressions of autophagy-related proteins (LC3B-I/LC3B-II and SQSTM1) in whole cellular (WCL) and nuclear extracts (nucleus) were detected by western blot analysis. (B) HepG2, DU145 and A549 cell lines transfected with EGFP-LC3 were treated with DOX (10 μM) for 24 h. Cells were stained with DAPI and the colocalization of EGFP-LC3 (green) and leaked DNA (blue) was examined by confocal microscopy. Scale bars: 10 μm. (C) Confocal microscopy images of HepG2, DU145 and A549 cell lines treated without or with DOX (10 μM) for 24 h after co-expression of mRFP-LC3 (red) and mGFP-LAMP1 (green) and staining with DAPI (blue). Scale bars: 10 μm.


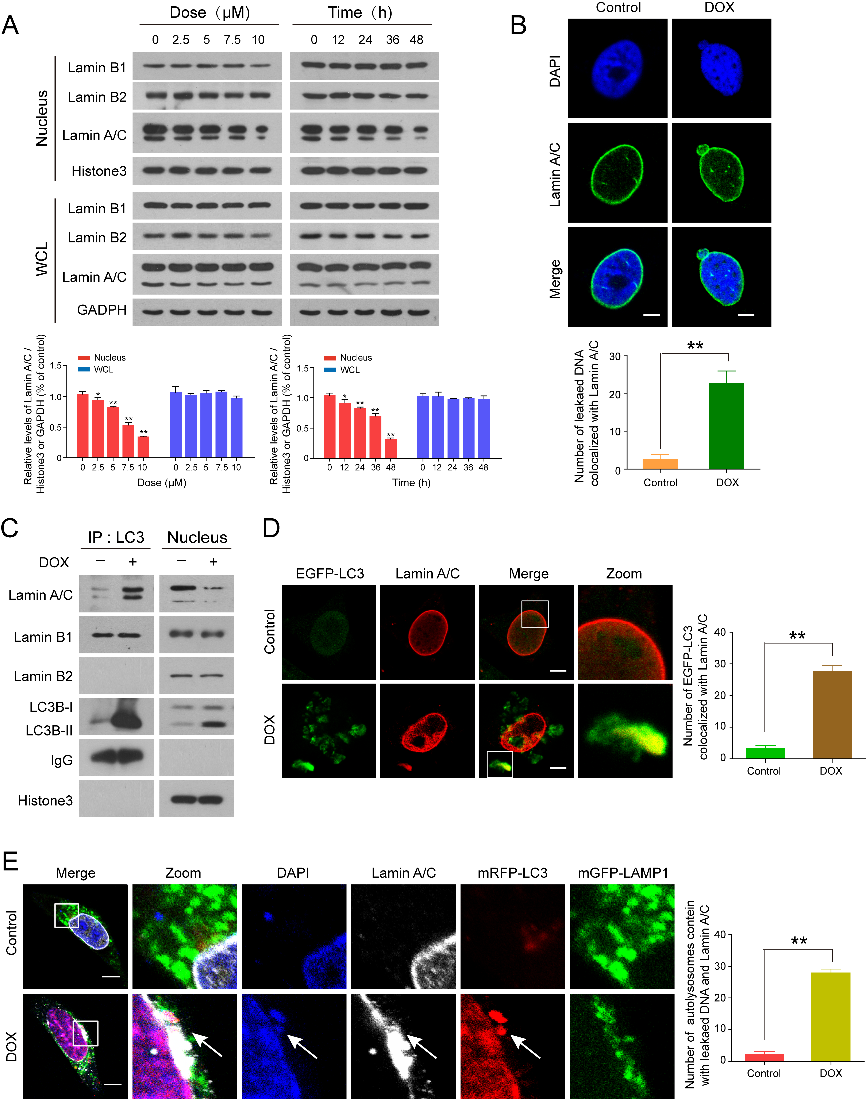


**Figure** **S3. LC3-lamin A/C interaction is required for nucleophagy.** MCF-7 cells were treated without or with DOX as indicated. (A) The expressions of nuclear proteins, lamin A/C, lamin B1, and lamin B2, in whole cellular (WCL) and nuclear extracts (nucleus) were detected by western blot. Comparison of the intensities were statistically estimated and represented as mean ± SD for three independent experiments (*P < 0.05, **P < 0.01). (B) The colocalization of lamin A/C (green) and leaked nuclear DNA (blue) was determined by confocal microscopy. Scale bars: 10 μm. The number of DAPI-stained particles with leaked nuclear DNA colocalized with lamin A/C was quantified from 30 cells in three independent experiments. Data was presented as mean ± S.D. (**P < 0.01). (C) After treatment with DOX (10 μM, 24 h), nuclear lysates were prepared and subjected to immunoprecipitation using anti-LC3, and the associated LC3B-I/LC3B-II, lamin A/C, lamin B1, and lamin B2 were determined by immunoblotting. (D) MCF-7 cells were transfected with EGFP-LC3, treated as in (B), and examined by confocal microscopy to determine the colocalization of EGFP-LC3 and lamin A/C (red). Scale bars: 10 μm. The number of EGFP-LC3 colocalized with lamin A/C was quantified from 30 cells in three independent experiments. Data was presented as mean ± S.D. (**P < 0.01). (E) Immunofluorescence analysis showed the colocalization of leaked DNA (blue) with mRFP-LC3(red), mGFP-LAMP1(green), and lamin A/C (white) in MCF-7 cells treated with DOX (10 μM, 24 h). Scale bars: 10 μm. The number of autolysosomes containing leaked DNA and lamin A/C was quantified from 50 cells in three independent experiments. Data was presented as mean ± S.D. (**P < 0.01).


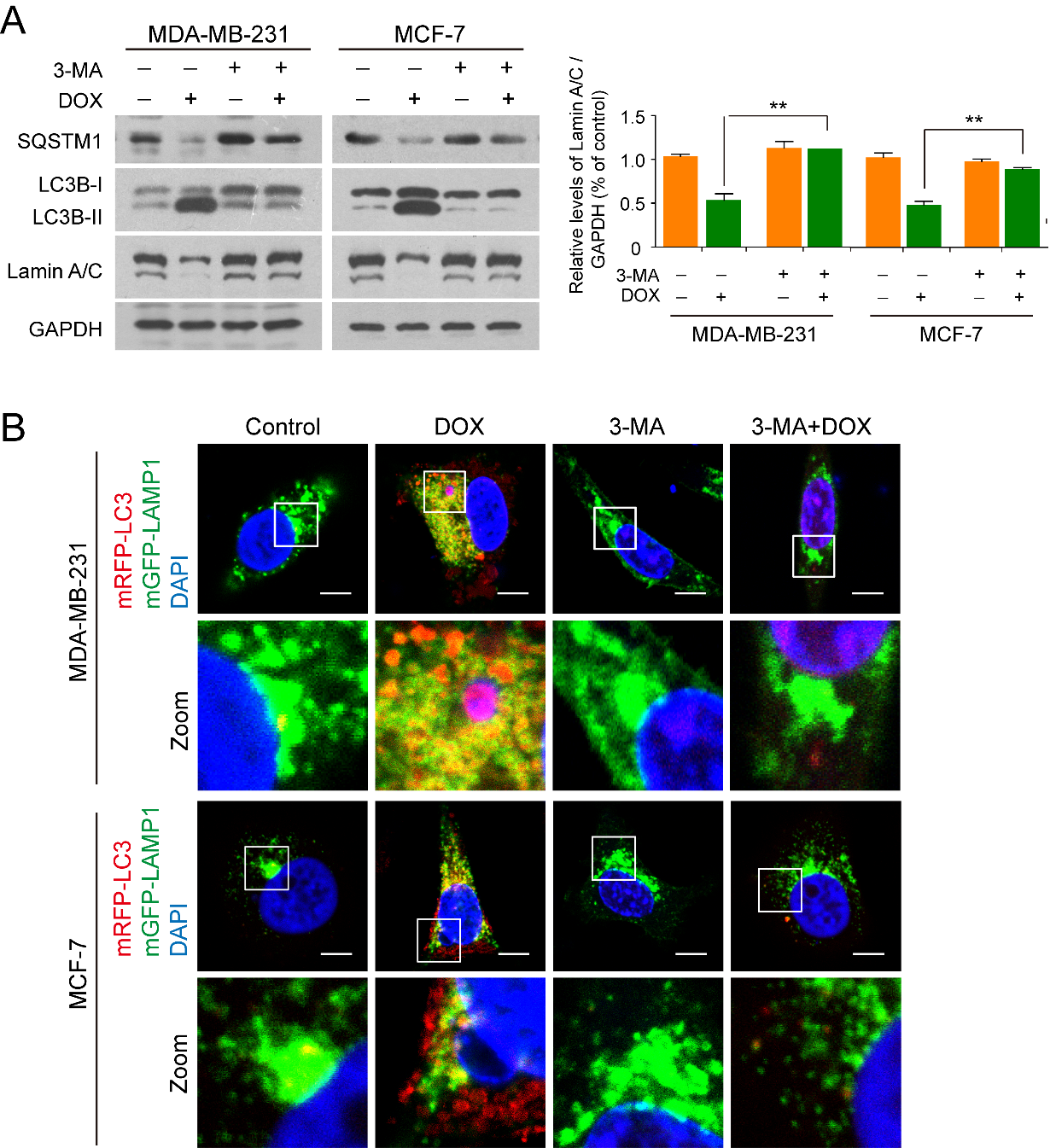


**Figure S4. Inhibiting autophagy impairs degradation of lamin A/C.** MDA-MB-231 and MCF-7 cells were exposed to DOX (10 μM) in the presence or absence of 3-MA (5 mM) for 24 h. (A) Nuclear extracts were prepared and subjected to western blot analysis using antibodies against to LC3B, SQSTM1, and lamin A/C. Data was presented as mean ± S.D. (**P < 0.01). (B) The localization of mRFP-LC3 (red), mGFP-LAMP1 (green) and leaked DNA (blue) was evaluated by confocal microscopy. Scale bars: 10 μm.


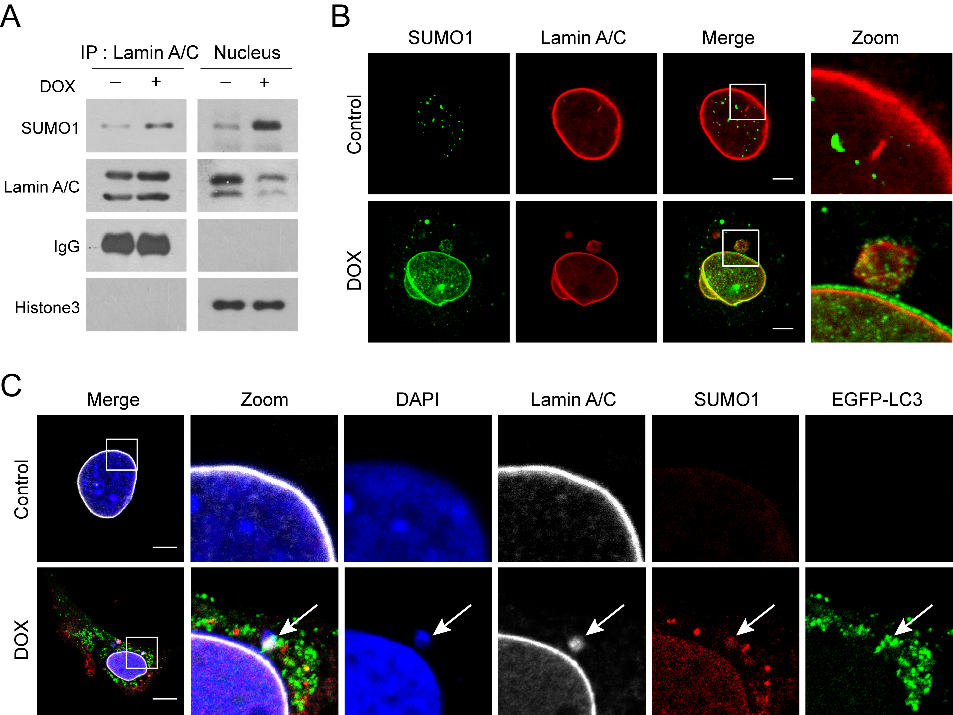


**Figure S5. Lamin A/C is SUMOylated in response to DNA damage.** MCF-7 cells were treated without or with DOX (10 μM) for 24 h. (A) Nuclear extracts were prepared and immunoprecipitated using anti-lamin A/C, and immunoblotted using anti-SUMO1. (B) Immunofluorescence analysis revealed colocalization of lamin A/C (red) and SUMO1 (green). Scale bars: 10 μm. (C) Immunofluorescence analysis showed a colocalization of SUMO1 (red) with EGFP-LC3 (green), lamin A/C (white) and leaked nuclear DNA (blue). Scale bars: 10 μm.


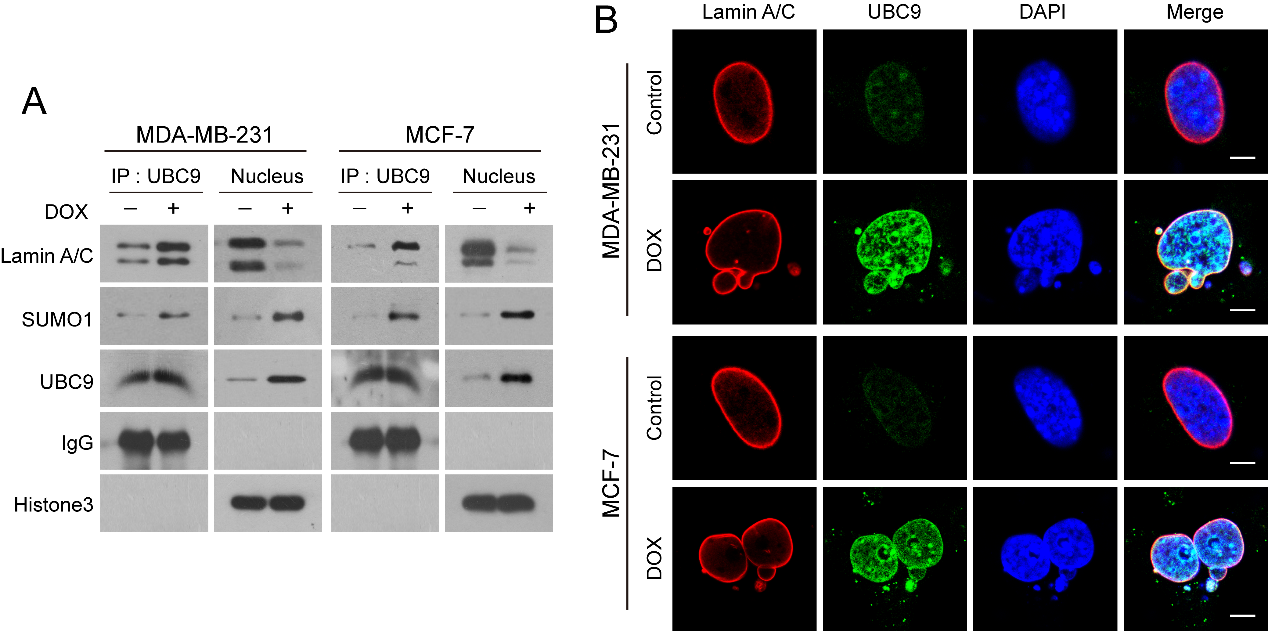


**Figure S6. UBC9 accumulates in nucleus in response to DNA damage.** MDA-MB-231 and MCF7 cells were treated with DOX (10 μM) for 24 h. (A) The expression of UBC9 in whole cellular (WCL) and nuclear extracts (nucleus) was detected by western blot analysis. (B) Confocal microscopy images showed accumulation of UBC9 in nucleus. Scale bars: 10 μm.
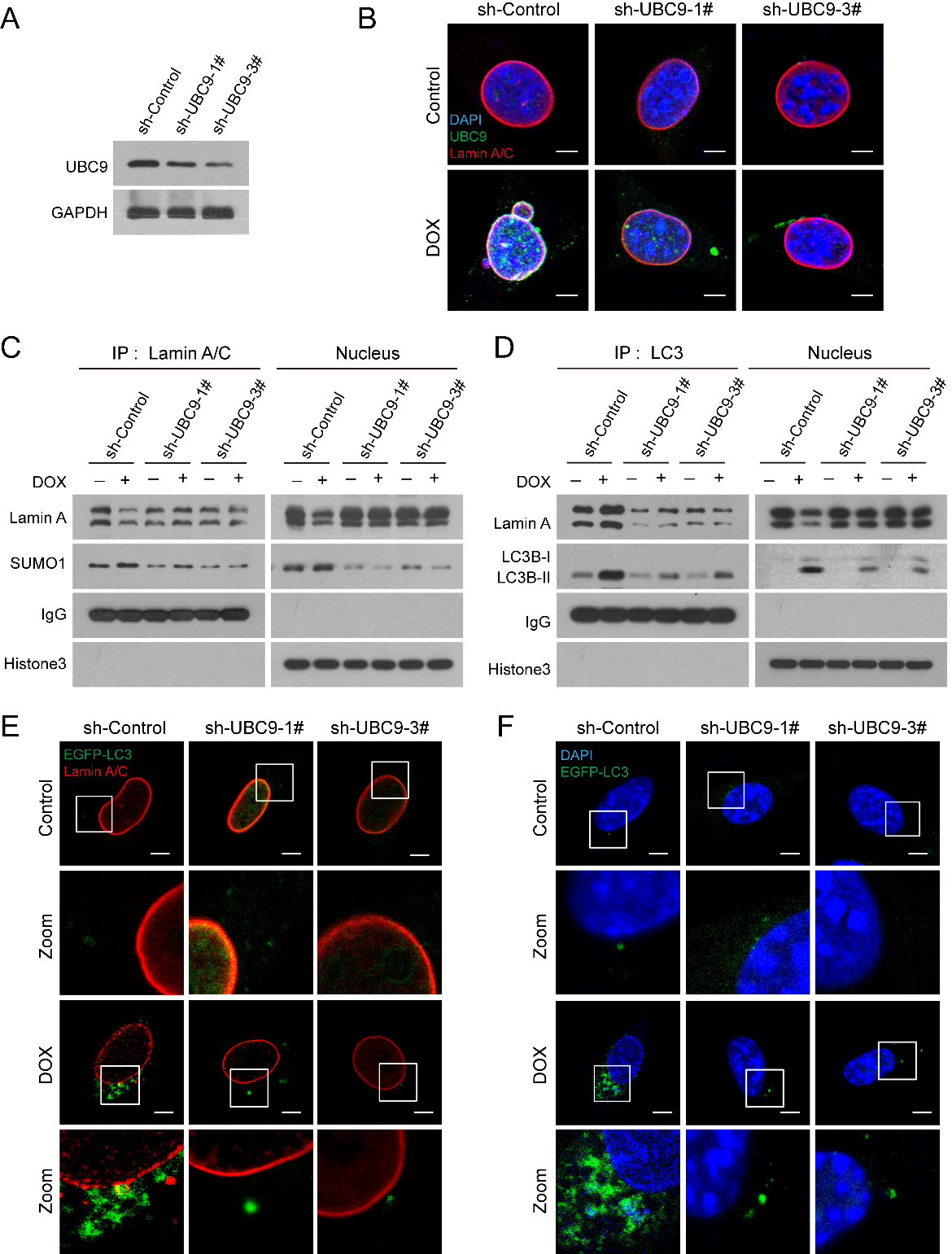


**Figure S7. Knockdown of UBC9 attenuates SUMOylation of lamin A/C and nucleophagy mediated by DNA damage.** MCF-7 cells stably expressing sh-Control and sh-UBC9 (1# and 3#) were treated with DOX (10 μM) for 24 h. (A) The expression of UBC9 in sh-Control and sh-UBC9 cells was detected by western blot. (B) Nuclear extracts were prepared, immunoprecipitated using anti-lamin A/C, and immunoblotted using anti-SUMO1. (C) The colocalization of lamin A/C (red), SUMO1 (green) and leaked DNA (blue) was determined by immunofluorescence. Scale bars: 10 μm. (D) Nuclear extracts were prepared, immunoprecipitated with anti-LC3, and immunoblotted with anti-lamin A/C. (E) The colocalization of EGFP-LC3 (green) and lamin A/C (red) was detected by immunofluorescence. Scale bars: 10 μm. (F) The colocalization of EGFP-LC3 (green) and leaked DNA (blue) was detected by immunofluorescence. Scale bars: 10 μm.
